# Video capsule endoscopy identifies severe small bowel lesions in a pediatric case of Henoch-Schönlein Purpura

**DOI:** 10.1055/a-2618-2420

**Published:** 2025-08-08

**Authors:** Youzhe Gong, Yanfei Chen, Meng Jin, Dan Zhu, Xuemei Zhong

**Affiliations:** 1 665434 Department of Gastroenterology, Childrenʼs Hospital Affiliated to Capital Institute Pediatrics, Beijing, China

A 12-year-old boy experienced recurrent abdominal pain for over 20 days after eating a cold hamburger. The pain, predominantly periumbilical, was accompanied by melena. Physical examination revealed periumbilical tenderness without any rash. Laboratory tests revealed an increase in WBC (23×10⁹/L), CRP (12.6 mg/L), ESR (18 mm/h), and D-dimer (2743 µg/L). Abdominal ultrasound revealed small bowel wall thickening, while the abdominal computed tomography scan showed no abnormalities. No significant lesions were found in gastroscopy and colonoscopy.

Video capsule endoscopy (VCE) was performed and revealed segmental mucosal inflammation in the jejunum and ileum, with multiple hemorrhagic spots, ecchymoses, erosions, irregular superficial ulcers, and focal areas of spontaneous bleeding ( Fig. 1 **a–d** , Video 1 ). Two days after the VCE, he developed hemorrhagic purpura on both lower limbs. A diagnosis of Henoch-Schönlein purpura (HSP) was made after ruling out other diseases. He was treated with methylprednisolone, resulting in significant relief of abdominal pain and resolution of melena. Follow-up abdominal ultrasound showed improvement in bowel wall thickening.

**Fig. 1:**
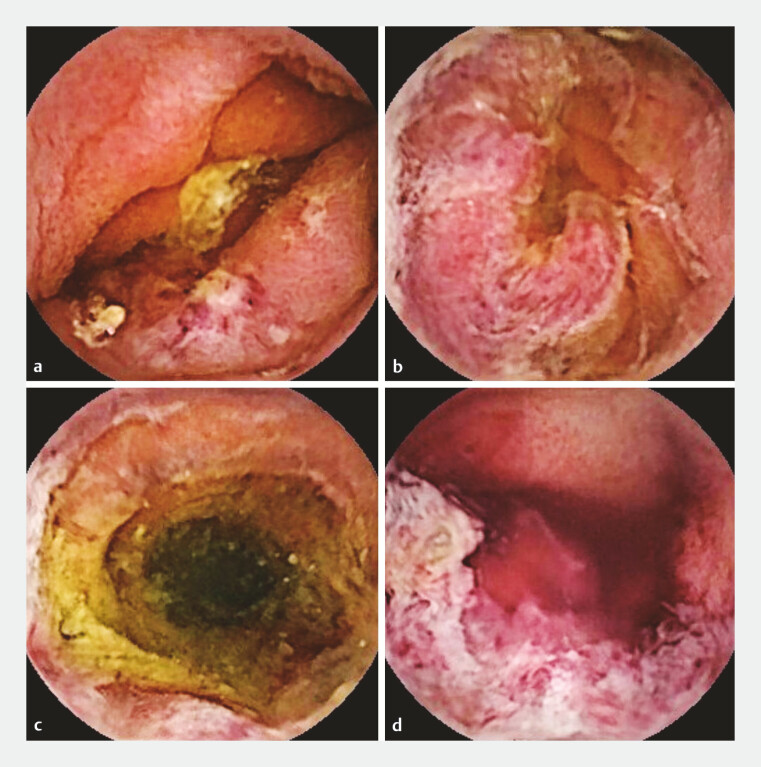
Video capsule endoscopy revealed multiple areas of purpuric erythema, irregular superficial ulcers, and focal areas of spontaneous bleeding in the jejunum and ileum. **a–c** Views from the jejunum. **d** View from the distal ileum.

Video capsule endoscopy identifies severe small bowel lesions in a pediatric case of Henoch-Schönlein purpura.Video 1

The patient additionally presented with hematuria, proteinuria, and hypertension. Renal biopsy findings were consistent with Henoch-Schönlein purpura nephritis type IIIb, and he then received mycophenolate mofetil treatment.

HSP is the most common systemic vasculitis in childhood, primarily occurring between the ages of 3 and 15 years 1 , yet diagnosing its small bowel involvement can be challenging. Reports on the use of VCE in children with HSP are rare. In 15%–35% of pediatric HSP cases, gastrointestinal symptoms precede the onset of purpura 1 2 . Studies have shown that the jejunum, ileum, and the distal part of the duodenum are the most commonly involved in gastrointestinal manifestations 3 . In this case, VCE revealed severe small intestinal lesions, whereas traditional endoscopic examinations failed to detect any abnormalities, providing a crucial clue for diagnosis.

Endoscopy_UCTN_Code_TTT_1AP_2AB

## Notes

Endoscopy E-Videos https://eref.thieme.de/e-videos
*E-Videos* is an open access online section of the journal *Endoscopy* , reporting on interesting cases and new techniques in gastroenterological endoscopy. All papers include a high-quality video and are published with a Creative Commons CC-BY license. Endoscopy E-Videos qualify for HINARI discounts and waivers and eligibility is automatically checked during the submission process. We grant 100% waivers to articles whose corresponding authors are based in Group A countries and 50% waivers to those who are based in Group B countries as classified by Research4Life (see: https://www.research4life.org/access/eligibility/ ). This section has its own submission website at https://mc.manuscriptcentral.com/e-videos .

## References

[1] Seiichi K Benjamin D Ayumu K Gastrointestinal manifestations and pathogenesis in childhood immunoglobulin A vasculitis Front Pediatr 2024 12 1.459394E6 10.3389/fped.2024.1459394 PMC11532042 39497734

[2] Fang Y Peng K Zhao H The characteristics of video capsule endoscopy in pediatric Henoch–Schönlein purpura with gastrointestinal symptoms Pediatr Rheumatol Online J 2020 18 84 33115491 10.1186/s12969-020-00471-4 PMC7592546

[3] Tanaka T Hiramatsu K Saito Y The usefulness of video capsule endoscopy in evaluating gastrointestinal manifestations of immunoglobulin A vasculitis Intern Med 2019 58 1979 1985 10.2169/internalmedicine.2097-18 30996162 PMC6702007

